# Targeting TCMR-associated cytokine genes for drug screening identifies PPARγ agonists as novel immunomodulatory agents in transplantation

**DOI:** 10.3389/fimmu.2025.1539645

**Published:** 2025-01-22

**Authors:** Lu Hu, Xiaohan Zhang, Weiqi Zhang, Shuai Jin, Jie Zhao, Jianming Zheng, Wenli Song, Zhongyang Shen

**Affiliations:** ^1^ The First Central Clinical School, Tianjin Medical University, Tianjin, China; ^2^ Research Institute of Transplant Medicine, School of Medicine, Nankai University, Tianjin, China; ^3^ School of Medicine, Nankai University, Tianjin, China; ^4^ Department of Renal Transplantation, Tianjin First Central Hospital, Nankai University, Tianjin, China; ^5^ NHC Key Laboratory for Critical Care Medicine, Tianjin First Central Hospital, Tianjin, China; ^6^ Key Laboratory of Transplant Medicine, Chinese Academy of Medical Sciences, Tianjin, China

**Keywords:** T-cell-mediated rejection, organ transplantation, cytokine genes, predictive model, drug repurposing, PPARγ agonists, rosiglitazone

## Abstract

**Objective:**

T cell-mediated rejection (TCMR) remains a significant challenge in organ transplantation. This study aimed to define a TCMR-associated cytokine gene set and identify drugs to prevent TCMR through drug repurposing.

**Methods:**

Gene expression profiles from kidney, heart, and lung transplant biopsies were obtained from the Gene Expression Omnibus (GEO) database. Differentially expressed genes (DEGs) between TCMR and non-TCMR groups were identified, and their intersection with cytokine-related genes yielded an 11-gene TCMR-associated cytokine gene set (TCMR-Cs). To evaluate the effectiveness of this gene set, a diagnostic predictive model was constructed using Lasso regression and multivariate logistic regression, with validation in independent datasets. Connectivity Map (CMap) analysis was employed to screen drugs targeting TCMR-Cs. Experimental validation of the identified drug was performed *in vitro* using T cell activation and Th1 differentiation assays, and *in vivo* in a mouse skin transplant model with survival analysis.

**Results:**

The TCMR-Cs exhibited outstanding predictive performance for TCMR, achieving an AUC of 0.99 in the training cohorts and maintaining strong performance in the test cohorts. CMap analysis identified peroxisome proliferator-activated receptor gamma (PPARγ) agonists as potential therapeutic candidates. Experimental validation showed that the PPARγ agonist rosiglitazone significantly suppressed T cell activation and reduced Th1 differentiation *in vitro* without cytotoxic effects. The combination of rosiglitazone and rapamycin significantly prolonged graft survival.

**Conclusions:**

This study defined a novel TCMR-associated cytokine gene set that effectively predicts TCMR and identified PPARγ agonists, which prevent TCMR and improve graft survival when combined with rapamycin.

## 1 Introduction

Organ transplantation is a life-saving treatment for patients with end-stage organ failure, significantly improving survival rates and quality of life (1). Kidney, heart, and lung transplantation procedures benefit tens of thousands of patients worldwide each year. However, long-term graft survival is often hindered by transplant rejection, among which T cell-mediated rejection (TCMR) remains one of the major challenges (2–4). TCMR arises from the activation of T cells through immunological synapses formed with antigen-presenting cells (APCs), such as dendritic cells and inflammatory myeloid cells. These interactions, mediated by the recognition of antigens by the T cell receptor (TCR), lead to interferon-gamma (IFNG)-driven inflammatory signaling pathways and the expression of inflammation-related genes, including IL2, IFNG, and ADAMDEC1. Together, these immune processes result in severe inflammation and graft dysfunction, highlighting the critical roles of T cells, dendritic cells, and macrophages in TCMR pathogenesis (5, 6).

Although immunosuppressive therapies, such as cyclosporine, tacrolimus, and mycophenolate mofetil, have demonstrated efficacy in reducing short-term rejection episodes (7), their long-term use is associated with substantial adverse effects, including infections, malignancies, hypertension, diabetes, and nephrotoxicity (8–12). These complications significantly impair patient quality of life and limit the applicability of current therapeutic regimens. Moreover, the diagnosis of TCMR relies heavily on invasive graft biopsies, which are prone to sampling errors, procedural complications, and subjective variations in histopathological evaluations (13, 14). These challenges underscore the critical need for innovative diagnostic tools and safer, more effective immunomodulatory interventions to address TCMR.

Cytokines, as key regulators of immune responses, represent promising candidates for improving both the diagnosis and treatment of TCMR. Cytokine-related genes play central roles in mediating immune dysregulation during TCMR, making them valuable targets for biomarker discovery and therapeutic development. To expand their translational potential, a broader understanding of cytokine dysregulation across different transplant types is necessary. A multi-organ perspective may uncover shared pathways underlying TCMR, facilitating the identification of universal targets for intervention.

Among potential therapeutic targets, the nuclear receptor peroxisome proliferator-activated receptor gamma (PPARγ) has received attention for its immunomodulatory and anti-inflammatory properties. Originally recognized for its role in regulating lipid and glucose metabolism in diseases such as type 2 diabetes and dyslipidemia (15, 16), PPARγ has now been implicated in modulating immune responses. PPARγ agonists, such as rosiglitazone, have been shown to attenuate T cell activation, alter cytokine production, and regulate macrophage polarization, thereby suppressing excessive inflammatory responses (17). These properties position PPARγ agonists as promising candidates for addressing immune dysregulation in TCMR and reducing graft rejection. However, their potential therapeutic value has yet to be fully explored in the context of organ transplantation.

In this study, we addressed the limitations of traditional diagnostic and therapeutic approaches to TCMR by identifying a novel TCMR-associated cytokine gene set (TCMR-Cs) through the integration of transcriptomic data from kidney, heart, and lung transplant rejection biopsies. This gene set provided valuable insights into the mechanisms underlying TCMR and served as the basis for both diagnostic and therapeutic innovations. A predictive model constructed using the TCMR-Cs demonstrated its utility in differentiating TCMR from non-TCMR patients, while drug repurposing analyses identified PPARγ agonists as promising immunomodulatory agents for TCMR prevention. Experimental validation further confirmed the efficacy of the PPARγ agonist rosiglitazone in modulating immune responses, including its ability to suppress T cell activation and prolong graft survival in combination with rapamycin. These findings lay a foundation for future precision medicine strategies in transplantation and highlight the potential of cytokine-based interventions to overcome the challenges of TCMR.

## 2 Materials and methods

### 2.1 Data acquisition and preprocessing

Gene expression microarray datasets for kidney (GSE192444) (18), heart (GSE150059) (19), and lung (GSE150156) (20) transplants were retrieved from the GEO database. All datasets were log2-transformed and normalized using the “limma” package in R to ensure consistency across datasets. The biopsy diagnosis for each sample was defined based on the original annotations provided in the corresponding dataset. In order to ensure a focused and accurate analysis, samples were regrouped into two categories: those with a biopsy diagnosis of “TCMR” were classified into the “TCMR” group, while those diagnosed with other conditions, such as “ABMR” or “No Rejection,” were classified into the “non-TCMR” group. To minimize potential bias and improve the specificity of the analysis, samples labeled as “Mixed Rejection,” “Possible TCMR,” “Borderline,” or “Possible ABMR” were excluded, as the inclusion of such heterogeneous or uncertain diagnoses could confound the identification of molecular features specific to TCMR (5).

### 2.2 DEG identification and functional enrichment analysis

DEGs were identified using the “limma” package in R. Linear models accompanied by empirical Bayes moderation were applied to stabilize variance estimates. DEGs were defined by a log-fold change (logFC) threshold of ±1 and a *p*-value < 0.05. Significant DEGs were then subjected to functional annotation using the “clusterProfiler” package. Gene Ontology (GO) analysis was performed to explore biological processes (BP), molecular functions (MF), and cellular components (CC) associated with these genes. Kyoto Encyclopedia of Genes and Genomes (KEGG) pathway analysis identified relevant biological pathways implicated by the DEGs. Both GO and KEGG analyses used the “org.Hs.eg.db” as the reference database. Visualization of GO and KEGG results was performed using bar plots or bubble charts.

### 2.3 Data visualization

Principal component analysis (PCA) was conducted using the “FactoMineR” and “factoextra” packages to visualize variance within datasets and achieve a clear separation between TCMR and non-TCMR groups. Heatmaps and volcano plots of DEGs were generated using the “EnhancedVolcano” and “pheatmap” packages, displaying differential expression patterns visually. To identify genes shared across kidney, heart, and lung datasets, Venn diagrams were generated using the “VennDiagram” R package. Additional visualizations were generated using the “ggplot2” package.

### 2.4 Definition of the TCMR-Cs

Upregulated DEGs shared across kidney, heart, and lung TCMR datasets were identified through intersection analysis. These shared DEGs were further cross-referenced with a cytokine-related gene set curated from the literature (21), resulting in 11 genes defined as the TCMR-Cs. Gene interaction networks among the TCMR-Cs were analyzed using GeneMANIA (http://genemania.org/) (22). This analysis included co-expression, physical interactions, and shared pathways to support the inclusion of these genes as a cohesive set.

### 2.5 Predictive model construction and validation

To validate the significance of the identified TCMR-Cs, a predictive model was constructed. Normalized gene expression data from the kidney (GSE192444), heart (GSE150059), and lung (GSE150156) datasets were combined to form the training cohort. Lasso regression for feature selection was implemented using the “glmnet” R package to address high-dimensional data. Cross-validation was utilized to determine the optimal lambda parameter, and lambda.1se was selected to balance model complexity and performance. Five hub genes (*CXCL13, IFNG, TNFSF13B, CCL3, CCL18*) were identified through this process. A multivariate logistic regression model was then constructed using the selected genes. Risk scores were calculated for each sample based on the following formula:


$$\text{Risk}\;\text{Score}=\sum_{\text{i}=1}^{\text{n}}\left(co\text{e}{\text{f}}_{\text{i}}\times \text{exp}{\text{r}}_{\text{i}}\right)$$

where $\text{co}ef_{i}$ represents the regression coefficient for gene i , and $expr_{i}$ indicates the expression level of gene i . The model’s performance was assessed through receiver operating characteristic (ROC) curve analysis. The risk score threshold was determined using the Youden index. Independent validation datasets from kidney (GSE98320) (23), heart (GSE124897) (24), and lung (GSE125004) (25) transplantation were used to evaluate the generalizability of the model. ROC curves and area under the curve (AUC) values were calculated for each test cohort. Given the class imbalance present in the datasets, additional performance metrics such as precision, recall, and F1 score were implemented to ensure a more thorough evaluation of the model’s predictive performance. These metrics were computed for both the training and independent test datasets to robustly validate the model.

### 2.6 Immune cell infiltration analysis

The CIBERSORT algorithm (26), utilizing the LM22 signature matrix, was employed to estimate the proportions of 22 immune cell types, providing insights into the immune landscape of the datasets. Differences in immune cell infiltration between TCMR and non-TCMR groups were visualized using box plots and heatmaps. Statistical significance was determined using Kruskal-Wallis tests, while correlation analysis was performed using the “Hmisc” package in R.

### 2.7 Drug screening

CMap (https://clue.io/) database (27) was used to identify small molecules with potential therapeutic effects based on connectivity scores that reflect the ability to reverse TCMR-associated gene expression. Among the top candidates, PPARγ receptor agonists were highlighted, and rosiglitazone was selected for further experimental validation.

### 2.8 Mouse skin transplantation model

Donor mice were 8-week-old male BALB/c mice, while recipient mice were 8–12-week-old male C57BL/6 mice. Donor mice were euthanized, and their tail skin was thoroughly disinfected with povidone-iodine. Tail skin was excised and trimmed into 1.0 × 1.0 cm² grafts. Recipient mice were anesthetized with isoflurane, and the hair on their dorsal region was shaved and disinfected with povidone-iodine. Once the disinfectant dried, a 1.0 × 1.0 cm² graft bed was prepared by excising the skin. The donor skin graft was placed onto the graft bed and secured at the four corners and edges using sutures. After surgery, the graft was protected with a sterile bandage, and the mice were housed in clean cages with heating pads to maintain body temperature until they fully recovered. The mice were divided into four groups: control group, rosiglitazone group, rapamycin group, and combination group (rosiglitazone + rapamycin). Starting on the day of transplantation, the mice received daily intraperitoneal injections of the respective treatments. The rapamycin group received 0.5 mg/kg/day, the rosiglitazone group received 10 mg/kg/day, and the combination group received both drugs at the same dosages. The control group received an equivalent volume of the solvent. Bandages were removed on postoperative day 7. Graft survival was assessed daily through macroscopic observation, and photographs were taken for analysis. Graft rejection was defined as ≥90% necrosis of the graft area.

### 2.9 Mouse CD4^+^ T cell culture

CD4^+^ T cells were activated in 96-well plates pre-coated with 5 μg/mL anti-mouse CD3ϵ antibody (BioLegend, Cat# 100340) in PBS, incubated overnight at 4°C. CD4^+^ T cells were isolated from the spleens of 6–8-week-old C57BL/6 mice using the CD4^+^ T Cell Isolation Kit (StemCell, Cat# 19765) following the manufacturer’s protocol. Spleens were homogenized through a 70 μm cell strainer to obtain single-cell suspensions, followed by red blood cell lysis using 0.84% ammonium chloride. Purified CD4^+^ T cells were resuspended in RPMI-1640 medium supplemented with 10% fetal bovine serum (FBS), 1% penicillin-streptomycin, 50 μM β-mercaptoethanol, 2 μg/mL anti-CD28 antibody (BioLegend, Cat# 102116), and 50 U/mL recombinant mouse IL-2 (BioLegend, Cat# 575402). The cells were seeded in the anti-CD3ϵ-coated plates and cultured at 37°C in a humidified atmosphere with 5% CO_2_. During the culture period, rosiglitazone was added to the medium at concentrations of 10 μM or 30 μM, or an equivalent volume of solvent was added as a control. After 24 hours of incubation, cells were harvested for flow cytometric analysis.

For Th1 differentiation, CD4^+^ T cells were cultured in RPMI-1640 medium supplemented with 10% fetal bovine serum, 1% penicillin-streptomycin, 50 μM β-mercaptoethanol, recombinant murine IL-12p70 (10 ng/mL, Propetech, Cat# 210-12-10UG), and neutralizing IL-4 antibody (10 μg/mL, BioLegend, Cat# 504122) under Th1-inducing conditions. The cells were seeded at a density of 1 × 10^6^ cells/mL in 96-well flat-bottom plates and cultured for 72 hours at 37°C in a humidified atmosphere with 5% CO_2_.

### 2.10 Flow cytometry analysis

To evaluate cell viability and death, CD4^+^ T cells treated with rosiglitazone (10 μM or 30 μM) or solvent control were first stained using the live-dead Zombie Aqua (BioLegend, Cat# 423102) according to the manufacturer’s instructions. Stained cells were then washed and resuspended in staining buffer (PBS containing 1% FBS and 0.1% sodium azide) for surface marker staining. For surface marker staining, cells were incubated with FITC-conjugated anti-mouse CD4 (ThermoFisher, Cat# 25-7021-82), APC-conjugated anti-mouse CD69 (BioLegend, Cat# 104513), and PE-conjugated anti-mouse CD25 (BioLegend, Cat# 102007) antibodies at 4°C in the dark for 30 minutes. After staining, cells were washed twice with cold staining buffer and resuspended in 300 μL PBS for analysis. For intracellular cytokine staining, cells were stimulated *in vitro* with 50 ng/mL PMA, 1 μg/mL ionomycin, and 10 μg/mL brefeldin A for 5 hours in a humidified 37°C incubator. Following stimulation, cells were fixed and permeabilized using Fixation Buffer (BioLegend, Cat# 420801) according to the manufacturer’s instructions. Intracellular IL-2 staining was performed using PE-Cy7-conjugated anti-mouse IL-2 antibody (ThermoFisher, Cat# 25-7021-82) at 4°C in the dark for 30 minutes.

For Th1 analysis, cells were collected following culture under Th1-inducing conditions, washed twice with cold PBS, and stained for CD4^+^ surface markers using FITC-conjugated anti-mouse CD4 antibody at 4°C in the dark for 30 minutes. After surface staining, intracellular cytokine staining was performed using the intracellular fixation and permeabilization kit (BioLegend, Cat# 420801). Subsequently, cells were stained for IFN-γ using anti-mouse IFN-γ-eFluor450 (ThermoFisher, Cat# 48-7311-82) at 4°C in the dark for 30 minutes. After washing, cells were resuspended in PBS with 1% FBS and analyzed using flow cytometry.

Flow cytometric analysis was conducted using a flow cytometer, and data were processed with FlowJo software (Version 10.8.1). The gating strategy was consistent across all samples. Debris and dead cells were excluded based on FSC/SSC properties during gating, and CD4^+^ T cells were selected for further analysis.

### 2.11 Statistical analysis

All statistical analyses were performed using R Software (version 4.3.0) and GraphPad Prism (version 9.5.1). Group comparisons were conducted using a two-tailed unpaired Student’s *t*-test for two groups or one-way analysis of variance (ANOVA) for comparisons among multiple groups. Graft survival in the skin transplantation experiments was analyzed using Kaplan-Meier survival curves and the log-rank test. The diagnostic performance of models was evaluated using ROC curves and AUC, with optimal cutoff points determined by the Youden index. Statistical significance was defined as a two-tailed *p* < 0.05.

## 3 Results

### 3.1 Identification and functional annotation of DEGs in TCMR patients across different organ transplants

To investigate the molecular mechanisms underlying TCMR across different transplanted organs, microarray data from kidney, heart, and lung transplant biopsy samples were retrieved from the GEO database and analyzed.

**Figure 1:**
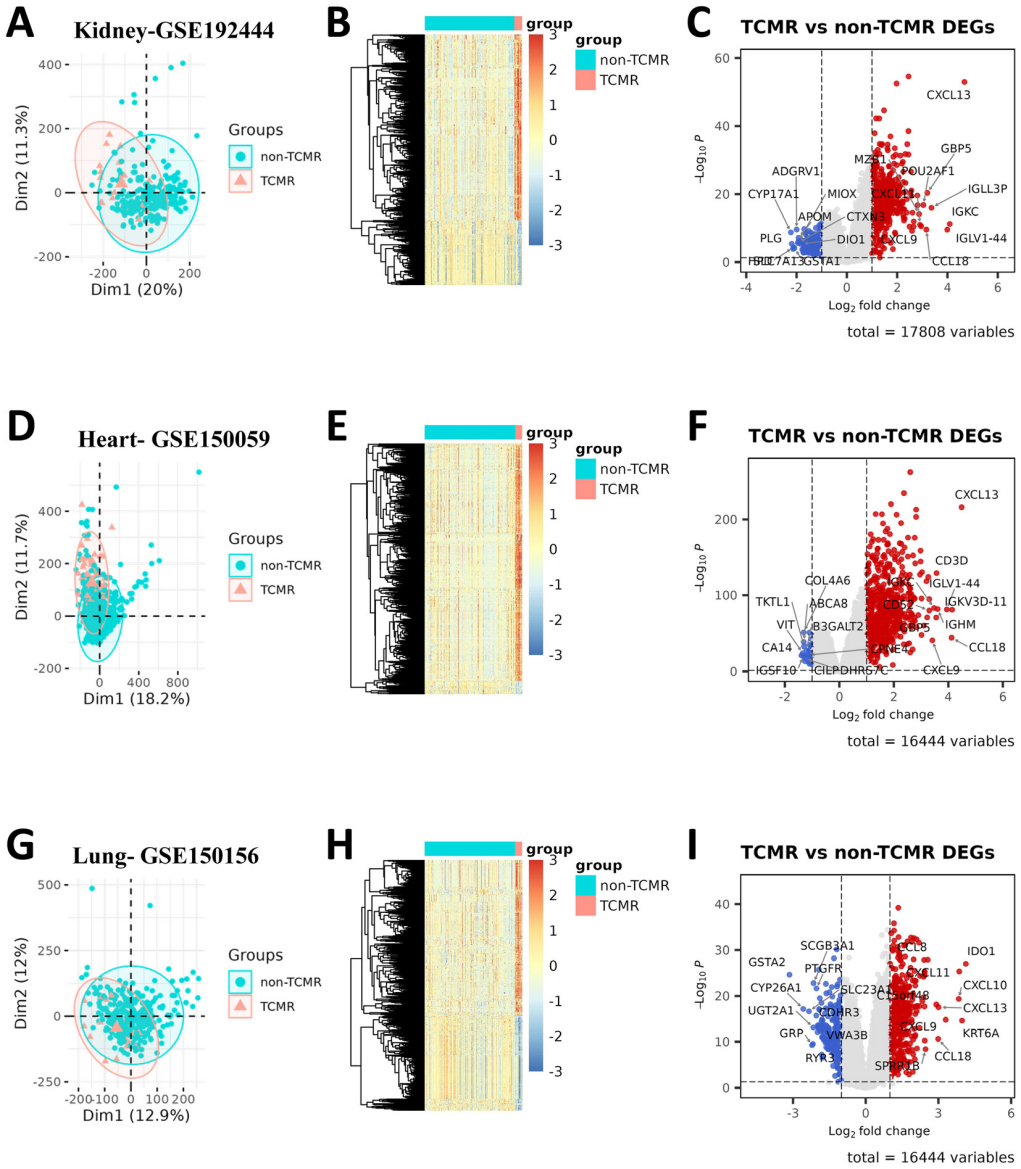
Differential gene expression between TCMR and non-TCMR groups across different transplant cohorts. PCA plots illustrate the separation of TCMR and non-TCMR samples in three transplant cohorts: renal transplant (GSE192444) **(A)**, heart transplant (GSE150059) **(D)**, and lung transplant (GSE150156) **(G)**. Each point represents an individual sample, with colors distinguishing the TCMR group from the non-TCMR group. Heatmaps **(B, E, H)** display the expression patterns of DEGs between TCMR and non-TCMR groups in the corresponding cohorts (GSE192444, GSE150059, and GSE150156). Rows correspond to DEGs, and columns represent individual samples. Volcano plots **(C, F, I)** present the results of DEG analysis between TCMR and non-TCMR groups for each cohort. Red dots indicate upregulated genes in the TCMR group, while blue dots indicate downregulated genes. The x-axis represents log2 fold change, and the y-axis represents -log10 p-value. Labeled genes denote the top 10 upregulated or downregulated genes with the highest absolute log2 fold changes.

In the kidney transplant cohort (GSE192444), which included 21 TCMR samples and 242 non-TCMR samples (**Figure 1A**), a total of 454 upregulated and 158 downregulated DEGs were identified (**Figures 1B, C**). Similarly, the heart transplant cohort (GSE150059), consisting of 76 TCMR samples and 1032 non-TCMR samples (**Figure 1D**), yielded 587 upregulated and 34 downregulated DEGs (**Figures 1E, F**). In the lung transplant cohort (GSE150156), including 23 TCMR samples and 291 non-TCMR samples (**Figure 1G**), 439 upregulated and 351 downregulated DEGs were identified (**Figures 1H, I**). These findings highlight the significant transcriptomic changes associated with TCMR in each organ system.

Functional annotation of the upregulated DEGs in each cohort using GO and KEGG pathway analyses revealed significant enrichment in immune-related biological processes and signaling pathways (**Supplementary Figures S1A–F**). Despite certain organ-specific differences in DEG profiles, key enriched pathways—such as “immune response-activating signaling pathway” and “immune receptor activity”—were consistently observed across kidney, heart, and lung transplants. This consistency highlights the shared immune processes underlying TCMR, regardless of the type of transplanted organ, further supporting the notion that TCMR is driven by conserved molecular mechanisms.

### 3.2 Shared molecular features and cytokine network analysis in TCMR patients

**Figure 2:**
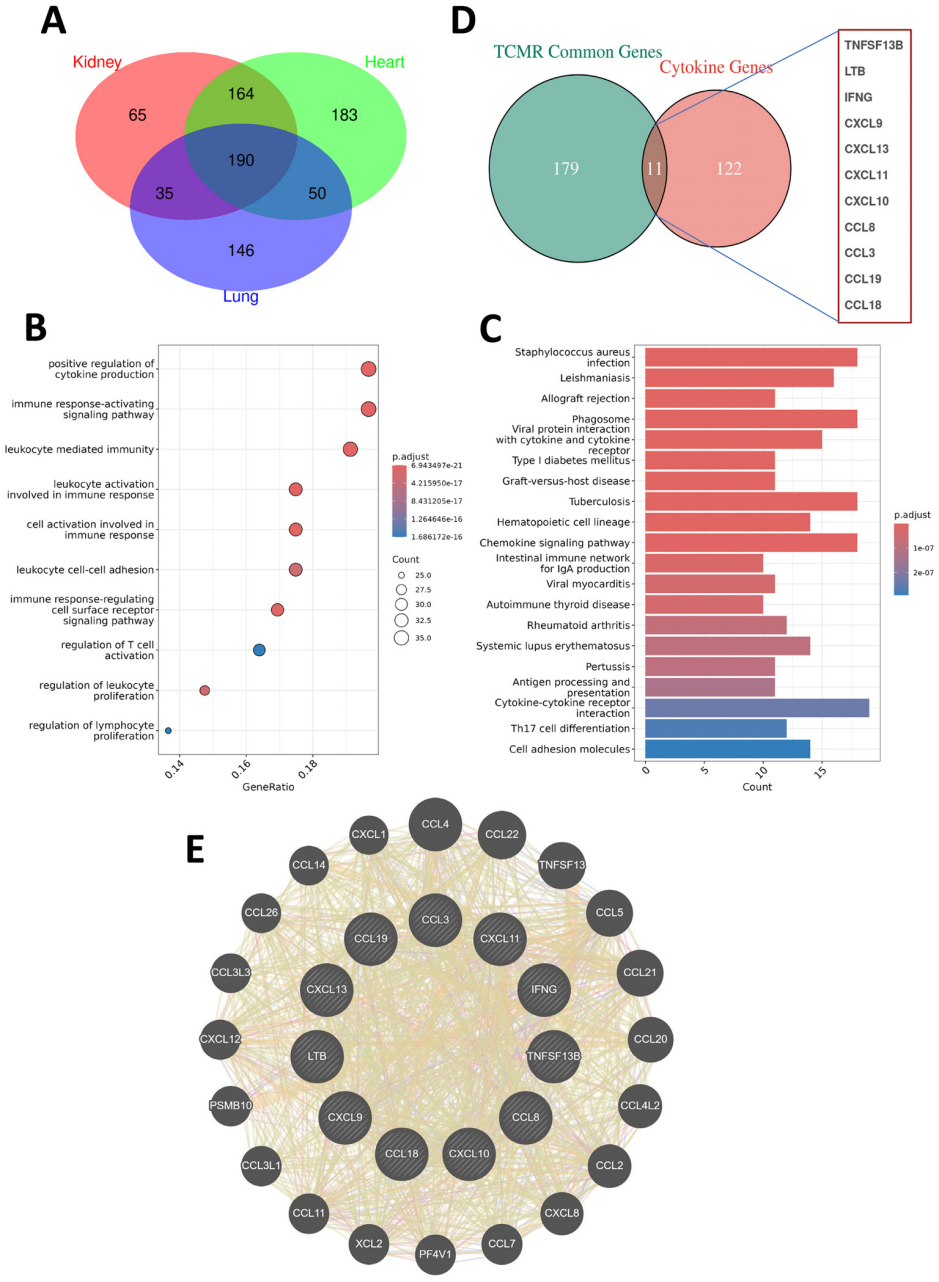
Shared gene characteristics and cytokine network analysis in TCMR patients. **(A)** A Venn diagram showing the commonly upregulated differentially expressed genes (CU-DEGs) among TCMR patients in renal transplant (GSE192444), heart transplant (GSE150059), and lung transplant (GSE150156) cohorts, identifying a total of 190 shared genes. **(B)** Bubble plot displaying the GO functional enrichment analysis of CU-DEGs. **(C)** Bar chart illustrating the KEGG pathway enrichment analysis of CU-DEGs. **(D)** A Venn diagram depicting the intersection between CU-DEGs and cytokine-related genes, identifying 11 TCMR-associated cytokine genes (TCMR-Cs). **(E)** PPI network of the TCMR-Cs constructed using the GeneMANIA database. The network reveals both direct and indirect interactions among the 11 key genes, along with additional functionally related genes.

To identify conserved molecular signatures across different transplanted organ types, we conducted an intersection analysis of the upregulated DEGs shared across kidney, heart, and lung TCMR cohorts. This approach identified 190 commonly upregulated DEGs (CU-DEGs) shared across the three organ types (**Figure 2A**). GO enrichment analysis of the CU-DEGs revealed significant associations with immune-related functional categories, including “positive regulation of cytokine production,” “immune response-activating signaling pathways,” “leukocyte activation involved in immune response,” “regulation of T cell activation,” and “leukocyte cell-cell adhesion” (**Figure 2B**). These results suggest that the shared DEGs play essential roles in driving immune activation and cell-cell interactions during TCMR. Further analysis using KEGG pathway enrichment demonstrated that CU-DEGs were prominently involved in pathways directly linked to transplant rejection, such as “allograft rejection,” “phagosome,” “graft-versus-host disease,” “hematopoietic cell lineage,” and “cytokine–cytokine receptor interaction” (**Figure 2C**). These enriched pathways highlight the interplay between innate and adaptive immune responses in TCMR pathogenesis and reinforce the critical role of a cytokine-driven inflammatory cascade in mediating transplant rejection.

By intersecting the identified CU-DEGs with a curated cytokine-related gene set, we further narrowed down a set of 11 TCMR-associated cytokine genes (TCMR-Cs): *CXCL13*, *IFNG*, *TNFSF13B*, *CCL3*, *CXCL11*, *CXCL9*, *CXCL10*, *CCL8*, *CCL18*, *LTB*, and *CCL19* (**Figure 2D**). These cytokine and chemokine genes are well-established as key regulators of immune responses and leukocyte recruitment, underscoring their importance in mediating immune dysregulation during TCMR. To explore the functional relationships among these TCMR-Cs, we constructed a protein-protein interaction (PPI) network using the GeneMANIA database. The resulting network revealed extensive direct and indirect interactions both within the cytokine gene set and with additional functional partners (**Figure 2E**). This PPI network illustrates the intricate and interconnected roles of these cytokines and chemokines in TCMR, emphasizing their collective contribution to immune activation, leukocyte recruitment, and inflammation. Collectively, these findings define a cytokine-driven axis of immune dysregulation in TCMR, which may serve as the basis for developing therapeutic strategies aimed at modulating cytokine activity during transplant rejection.

### 3.3 Diagnostic predictive model for TCMR using lasso and logistic regression

To further explore the biological significance and diagnostic potential of the previously identified 11 TCMR-Cs, we used their expression profiles to construct a predictive model for TCMR. This approach aimed to evaluate whether these cytokine genes collectively could reliably distinguish TCMR patients from non-TCMR patients across multiple organ transplant types.

**Figure 3:**
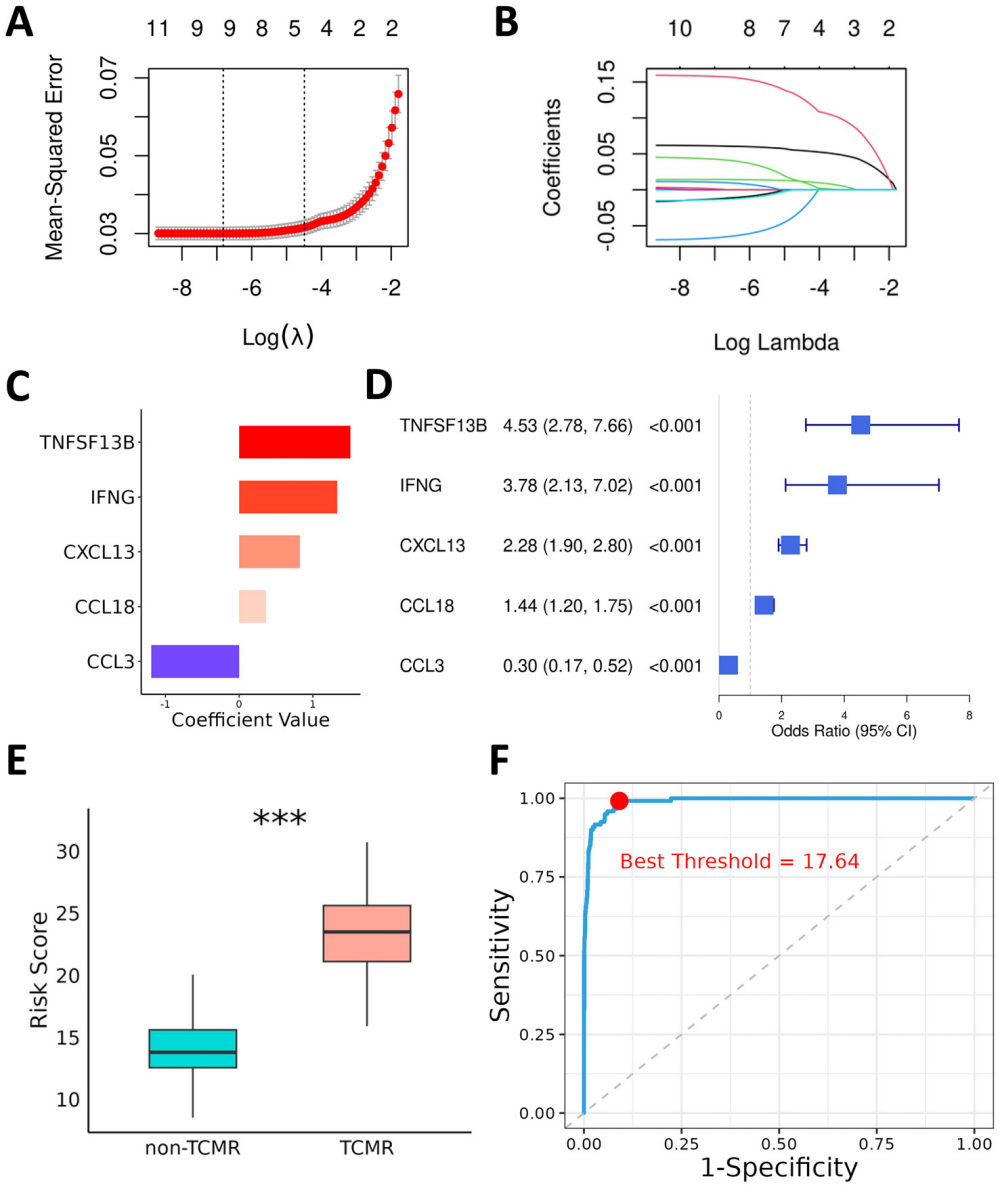
Diagnostic Model for TCMR Using Lasso and Logistic Regression. **(A)** Cross-validation plot for the Lasso regression, showing the trend of mean squared error (MSE) with the logarithmically transformed lambda parameter during cross-validation. **(B)** Coefficient path plot of the Lasso regression, illustrating the shrinkage of candidate gene coefficients toward zero as the regularization parameter lambda increases. At the selected lambda value (lambda.1se), five key genes were retained for constructing the prediction model. **(C)** Logistic regression coefficients of the TCMR-Hubs. **(D)** Forest plot summarizing the odds ratios (OR) and 95% confidence intervals (CIs) of the five key genes identified by logistic regression. **(E)** Box plot depicting the risk scores for each sample in the training cohort, calculated based on the regression coefficients of the five key genes. Significant differences in risk scores are shown between the TCMR and non-TCMR groups. **(F)** ROC curve of the risk scores, with the red dot indicating the optimal threshold determined by the Youden index for distinguishing TCMR and non-TCMR groups. ***P< 0.001.

The 11 TCMR-Cs were used as input features for modeling, and transcriptomic data from the kidney (GSE192444), heart (GSE150059), and lung (GSE150156) transplant cohorts were integrated to form a comprehensive multilayer dataset. Patients were categorized into TCMR (120 samples) and non-TCMR (1565 samples) groups based on clinical outcomes. To select the most informative genes and construct the optimal model, Lasso regression analysis was performed (**Figure 3A**). After tuning the parameters using the lambda.1se selection criterion, five genes—*CXCL13*, *IFNG*, *TNFSF13B*, *CCL3*, and *CCL18*—were identified as the key predictors for the final model (**Figure 3B**). These five genes are referred to as TCMR Core Hub Genes (TCMR-Hubs).

To examine the specific contributions of these TCMR-Hubs to the predictive model, multivariate logistic regression was conducted, revealing their individual regression coefficients and highlighting their predictive importance. The coefficients for the selected genes were as follows: *CXCL13* (0.83), *IFNG* (1.33), *TNFSF13B* (1.51), *CCL3* (-1.19), and *CCL18* (0.36) (**Figure 3C**). The corresponding odds ratios (ORs) and 95% confidence intervals (CIs) further validated their diagnostic relevance (**Figure 3D**). Using these coefficients, risk scores for all patients in the training cohort were calculated, providing a quantitative basis for stratifying patients by TCMR risk. Comparative analysis of risk scores between TCMR and non-TCMR patients showed a pronounced separation, with TCMR patients exhibiting significantly higher scores (**Figure 3E**). ROC curve evaluation confirmed the predictive performance of the model, achieving an exceptional AUC of 0.99 in the training cohort (**Figure 3F**). Using the Youden index, the optimal risk score threshold for classification was determined to be 17.64, effectively distinguishing high-risk patients from low-risk individuals. These results indicate the high diagnostic accuracy of the model and emphasize the biological and predictive importance of the five TCMR-Hubs in capturing immune features associated with TCMR.

### 3.4 Validation of the TCMR predictive model

**Figure 4:**
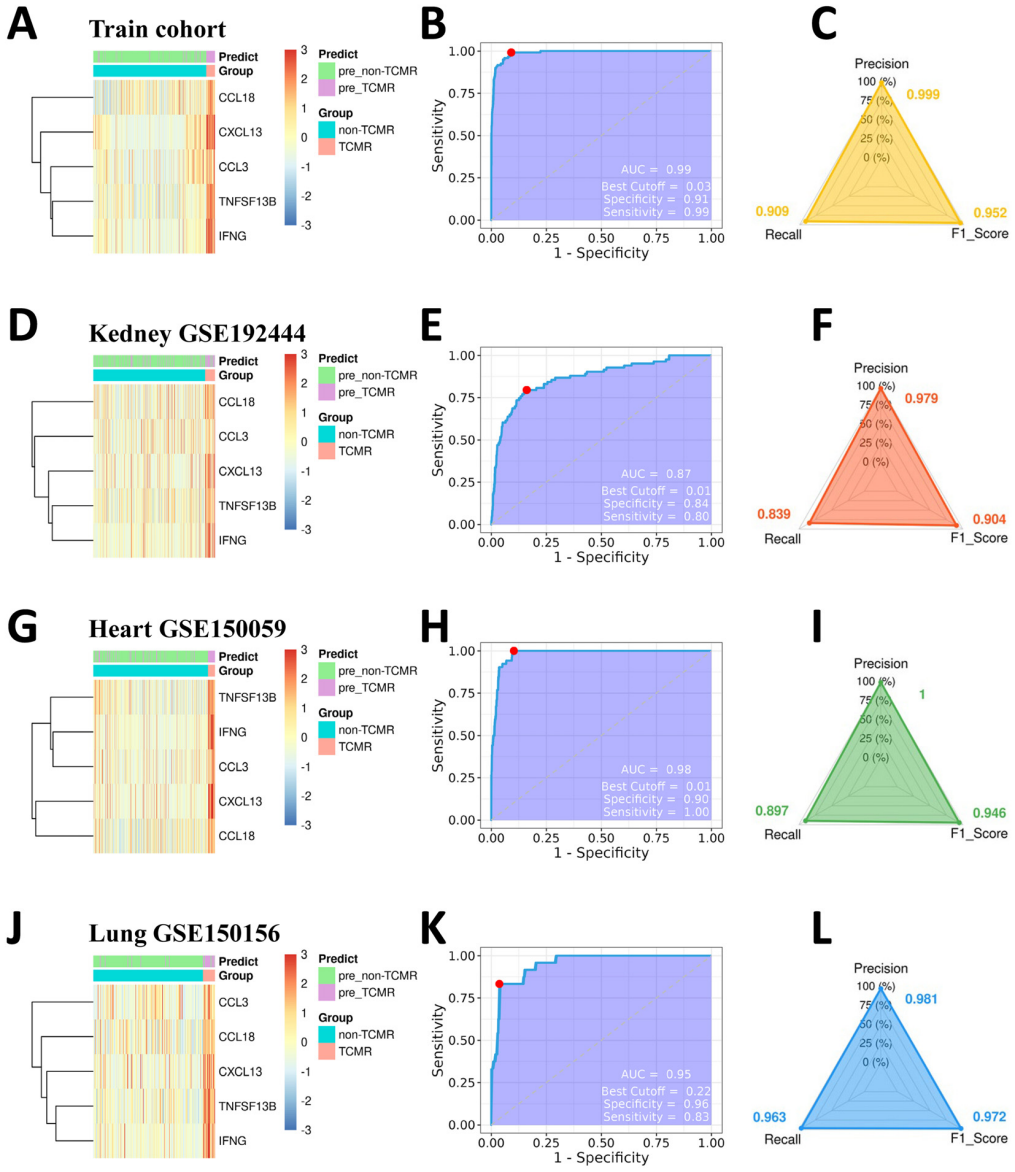
Evaluation of the predictive performance of the TCMR diagnostic model. Heatmaps display the expression patterns of TCMR-Hubs in the TCMR and non-TCMR groups across the training cohort **(A)**, renal transplant testing cohort (GSE98320, **(D)**), heart transplant testing cohort (GSE124897, **(G)**), and lung transplant testing cohort (GSE125004, **(J)**). Receiver operating characteristic (ROC) curves for the prediction model are shown for the training cohort **(B)**, renal transplant testing cohort (GSE98320, **(E)**), heart transplant testing cohort (GSE124897, **(H)**), and lung transplant testing cohort (GSE125004, **(K)**). Each ROC curve includes the AUC, optimal cutoff point, specificity, and sensitivity. Performance metrics, including precision, recall, and F1 score, are shown for the training cohort **(C)**, renal transplant testing cohort **(F)**, heart transplant testing cohort **(I)**, and lung transplant testing cohort **(L)**.

To validate the robustness and generalizability of the predictive model, we first evaluated its performance within the training cohort. The distinct expression profiles of the five TCMR-Hubs between TCMR and non-TCMR samples provided a solid basis for accurate classification (**Figure 4A**). At an optimal cutoff of 0.03, the model demonstrated excellent sensitivity (0.99) and specificity (0.91) in the training cohort, with an outstanding AUC of 0.99 (**Figure 4B**). Moreover, additional metrics were utilized to provide a more nuanced assessment of the model’s diagnostic performance in the training data. Specifically, the precision, recall, and F1 score of the model were 0.999, 0.909, and 0.952 (**Figure 4C**), respectively, indicating a strong balance between minimizing false positives and maximizing detection of TCMR cases. These results reinforced the ability of the model to reliably identify TCMR within the development dataset.

The predictive capability of the model was then validated across independent test cohorts, representing kidney (GSE98320), heart (GSE124897), and lung (GSE125004) transplants, to confirm its applicability across diverse transplant types. Additional performance metrics were calculated for these test datasets to ensure comprehensive evaluation. In the kidney test cohort, the model displayed strong diagnostic performance, achieving an AUC of 0.87 (**Figures 4D, E**), with precision, recall, and F1 score of 0.979, 0.839, and 0.904 (**Figure 4F**), respectively. Similarly, in the heart test cohort, the model maintained exceptional accuracy with an AUC of 0.98 (**Figures 4G, H**), combined with a precision of 1.000, recall of 0.897, and an F1 score of 0.946 (**Figure 4I**). Lastly, the lung transplant test cohort achieved high predictive power with an AUC of 0.95 (**Figures 4J, K**), alongside precision, recall, and F1 score values of 0.981, 0.963, and 0.972 (**Figure 4L**), respectively. These additional metrics not only confirmed the strong diagnostic capabilities of the model but also highlighted its robustness in handling imbalanced datasets, as evidenced by consistently high precision and balanced recall.

Collectively, these findings indicate that the TCMR predictive model based on the TCMR-Cs gene set can robustly and accurately assess TCMR risk across various organ transplant types. By integrating transcriptomic data and focusing on a core set of cytokine genes, this model demonstrates potential clinical utility for the early detection of TCMR.

### 3.5 Immune infiltration characteristics in TCMR patients

**Figure 5:**
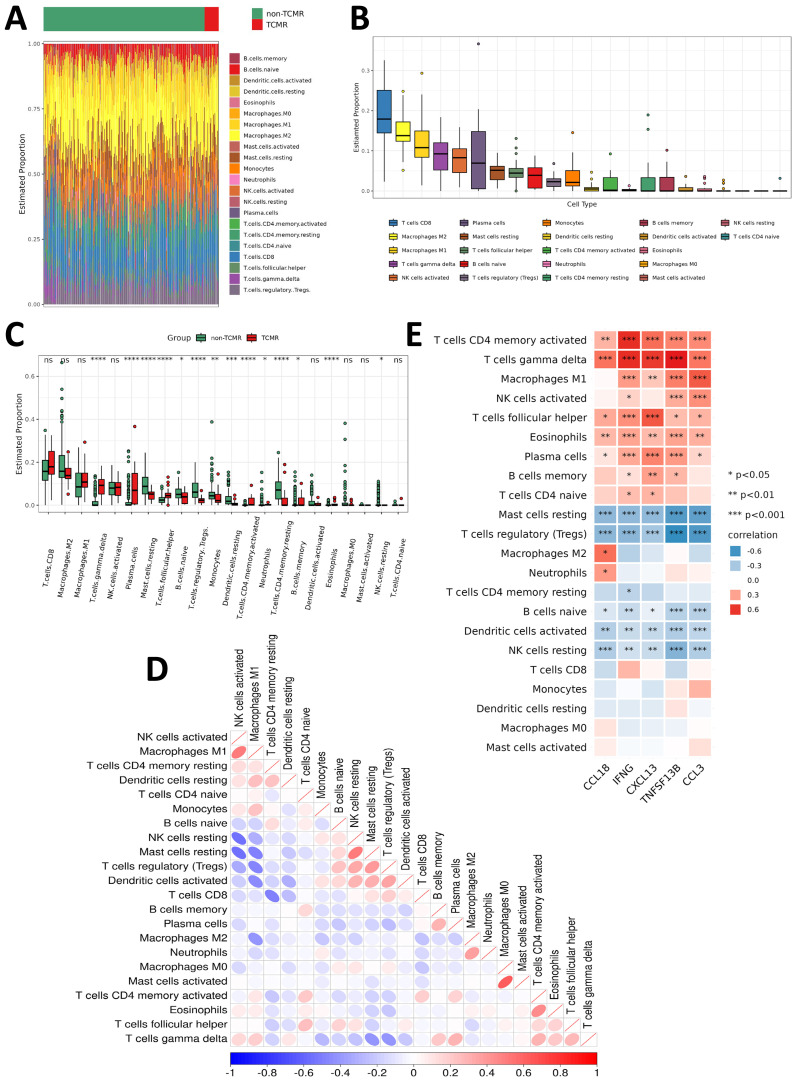
Immune infiltration characteristics of TCMR patients. **(A)** Heatmap showing the infiltration levels of various immune cells in the TCMR and non-TCMR groups within the renal transplant cohort (GSE192444). **(B)** Box plot ranking the abundance of immune cell infiltration in the TCMR group within the renal transplant cohort (GSE192444). **(C)** Box plot comparing the infiltration levels of immune cells between the TCMR and non-TCMR groups within the renal transplant cohort (GSE192444). **(D)** Correlation matrix illustrating the relationships among different immune cells in the TCMR group. Blue indicates negative correlations, while red indicates positive correlations, with deeper colors representing stronger correlations. **(E)** Correlation analysis between TCMR-Hubs and immune cell infiltration levels in the TCMR group. Blue indicates negative correlations, while red indicates positive correlations, with deeper colors representing stronger correlations. *P < 0.05, **P < 0.01, ***P < 0.001. *p<0.05, **p<0.01, ***p<0.001, ****p<0.0001 and non-significant values (ns) indicate p≥0.05.

To investigate the immune microenvironment of TCMR, immune cell infiltration levels were analyzed in TCMR and non-TCMR patients across kidney, heart, and lung transplant cohorts. Despite organ-specific differences, the TCMR groups consistently demonstrated elevated infiltration of several immune cell subsets, including CD8^+^ T cells, activated NK cells, M1 macrophages, M2 macrophages, γδ T cells, follicular helper T cells (Tfh cells), naive B cells, and plasma cells (**Figures 5A, B**; **Supplementary Figures S2A–D**).

Organ-specific differences in immune infiltration were observed when comparing TCMR patients to non-TCMR patients. In the kidney transplant cohort, infiltration of M1 macrophages, γδ T cells, plasma cells, and Tfh cells was significantly increased (**Figure 5C**). Among heart transplant TCMR patients, higher infiltration levels of CD8^+^ T cells, M1 macrophages, γδ T cells, and Tfh cells were observed (**Supplementary Figure S2E**). Similarly, lung transplant TCMR patients showed elevated infiltration of M1 macrophages, activated NK cells, γδ T cells, and activated memory CD4^+^ T cells (**Supplementary Figure S2F**). These observations highlight both shared and organ-specific immune mechanisms in TCMR. In contrast, several immune subsets were consistently reduced across all three organ types in the TCMR group, including regulatory T cells (Tregs) and resting mast cells. Moreover, M2 macrophages displayed lower infiltration levels in both kidney and heart TCMR cohorts (**Figure 5C**; **Supplementary Figures S2E, F**). This indicates a shift in the immune environment of TCMR patients towards a pro-inflammatory state, characterized by an imbalance between immune activation and regulation.

Correlational analysis between immune cell subsets revealed strong positive associations between certain immune populations, such as M1 macrophages and activated NK cells (**Figure 5D**; **Supplementary Figures S3A, C**). Furthermore, the relationship between the five TCMR-Hubs and immune cell infiltration was examined. Positive correlations were identified between TCMR-Hubs and immune-activated subsets, including γδ T cells, activated memory CD4^+^ T cells, plasma cells, activated NK cells, M1 macrophages, and Tfh cells. Conversely, TCMR-Hubs were negatively correlated with immune-regulatory or resting subsets, such as Tregs, resting NK cells, resting mast cells, naive B cells, and resting memory CD4^+^ T cells (**Figure 5E**; **Supplementary Figures S3B, D**).

These findings emphasize the critical role of specific immune subsets, particularly pro-inflammatory and effector cells, in the progression of TCMR. The observed negative correlations with regulatory immune populations further underscore the imbalance within the immune microenvironment in TCMR. These results suggest that modulating key immune subsets may provide a therapeutic strategy for mitigating TCMR.

### 3.6 Immunomodulatory effects of the PPARγ agonist rosiglitazone in TCMR

To identify potential therapeutic agents for TCMR, we focused on utilizing the TCMR-Cs to explore targeted treatment strategies. Using the 11 TCMR-Cs and the top 10 upregulated DEGs in TCMR patients, a drug-repurposing analysis was conducted through the CMap database. The analysis yielded a ranked list of small-molecule compounds with potential relevance to TCMR, based on their ability to reverse the TCMR-associated transcriptional signature. From the top 100 compounds showing negative CMap scores, the most frequent mechanisms of action included “PPAR receptor agonist,” “serotonin receptor antagonist,” “acetylcholine receptor antagonist,” “carbonic anhydrase inhibitor,” and “HDAC inhibitor” (**Supplementary Figure S4**). Among these, PPAR receptor agonists stood out due to their established anti-inflammatory and immunomodulatory properties. To validate their therapeutic potential, rosiglitazone, a PPARγ agonist, was selected for *in vitro* and *in vivo* functional experiments.

**Figure 6:**
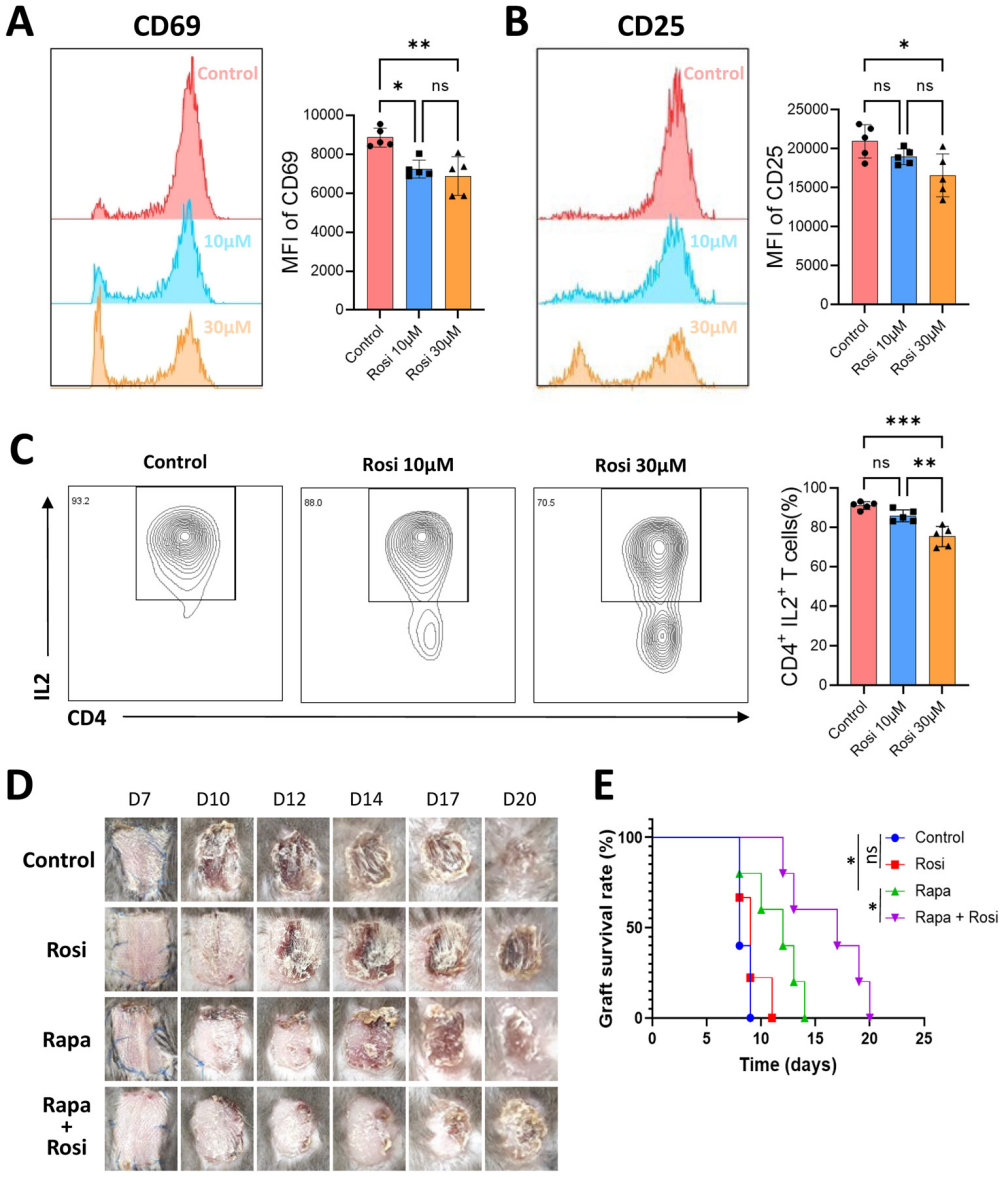
Immunoregulatory effects of the PPARγ agonist rosiglitazone in TCMR. CD4+ T cells were isolated from mouse spleens, activated *in vitro*, and treated with 10 μM or 30 μM rosiglitazone (Rosi) or an equivalent volume of solvent control for 24 hours. Flow cytometry was used to measure the mean fluorescence intensity (MFI) of CD69 **(A)** and CD25 **(B)** in each group. **(C)** Proportion of IL-2-positive CD4+ T cells measured by flow cytometry after 24 hours of culture. **(D)** Representative photographs of skin grafts from four groups of mice: control group, rosiglitazone group, rapamycin group (Rapa), and rapamycin combined with rosiglitazone group. **(E)** Survival curves of skin grafts from the four experimental groups. *p<0.05, **p<0.01, ***p<0.001 and ns indicate p≥0.05.

To evaluate its immunomodulatory capacity, CD4^+^T cells were isolated from mouse spleens, activated with anti-CD3, anti-CD28, and IL-2, and treated *in vitro* with rosiglitazone at concentrations of 10 μM and 30 μM. After 24 hours of treatment, flow cytometry analysis revealed that rosiglitazone significantly inhibited CD4^+^T cell activation, as evidenced by reduced expression of activation markers CD69 and CD25 (**Figures 6A, B**), and suppressed functional activity by significantly reducing IL-2 production (**Figure 6C**). Moreover, at both concentrations, no significant differences in cell viability and death were observed between the rosiglitazone-treated and control groups (**Supplementary Figure S5A**), indicating that rosiglitazone effectively modulates immune responses without inducing cytotoxicity under the tested conditions. Additionally, the effect of rosiglitazone on Th1 differentiation was investigated. CD4^+^T cells were isolated and cultured in Th1-inducing conditions (recombinant IL-12 and anti-IL-4 antibody) for 72 hours in the presence or absence of rosiglitazone. Flow cytometry analysis revealed that rosiglitazone treatment significantly reduced the proportion of Th1 cells, as indicated by a decreased percentage of CD4^+^IFN-γ^+^cells compared to the untreated Th1-differentiated control (**Supplementary Figure S5B**). These results suggest that rosiglitazone not only inhibits T cell activation but also suppresses the differentiation of pro-inflammatory Th1 cells.

The therapeutic potential of rosiglitazone was further assessed *in vivo* using a murine skin transplant rejection model. BALB/c mouse skin was transplanted onto the backs of C57BL/6 mice, and the animals were divided into four groups: control, rosiglitazone (10 mg/kg/day), rapamycin (50 mg/kg/day), and a combination of rosiglitazone and rapamycin (**Figure 6D**). In the rosiglitazone-only group, monotherapy did not significantly prolong graft survival compared to the control group. In contrast, rapamycin-treated mice exhibited extended graft survival. Importantly, the combination therapy of rapamycin and rosiglitazone demonstrated a synergistic effect, significantly prolonging graft survival compared to rapamycin alone (**Figure 6E**).

These results highlight the immunomodulatory potential of PPARγ agonists in TCMR. Rosiglitazone effectively suppressed T cell activation *in vitro*, reduced Th1 differentiation, and demonstrated no cytotoxic effects on T cells under the tested conditions, supporting its safety and efficacy as an immunomodulatory agent. *In vivo*, rosiglitazone enhanced graft survival when combined with rapamycin, as evidenced by extended survival times, suggesting a synergistic mechanism. These findings provide robust evidence supporting the repurposing of PPARγ agonists as adjunctive therapies for TCMR. Furthermore, they underscore the importance of combining targeted immunomodulators with existing immunosuppressive regimens to optimize outcomes in transplantation medicine.

## 4 Discussion

This study defines a novel TCMR-associated cytokine gene set and highlights its potential in both understanding the pathogenesis of TCMR and facilitating drug repurposing efforts to address this significant clinical challenge in organ transplantation. By integrating multi-organ transcriptomic data, we established an organ-agnostic approach to investigate key immune mechanisms underlying TCMR and proposed innovative therapeutic strategies based on these findings. Early detection of TCMR and the identification of targeted interventions are critical for improving long-term transplant outcomes (28), especially since current immunosuppressive therapies often result in severe complications such as infections, organ toxicity, and malignancy with prolonged use (7, 29). Responding to the urgent demand for safer and more sustainable treatments, this study offers a new framework for identifying actionable biomarkers, constructing predictive models, and exploring drug repurposing opportunities in transplantation medicine.

By focusing on the shared immune characteristics of TCMR across kidney, heart, and lung transplantation, we identified a cytokine-driven gene signature with significant translational potential. The intersection of TCMR-related genes with cytokine-associated gene sets yielded a concise and relevant gene signature (TCMR-Cs) that provides a foundation for understanding common immune mechanisms and identifying therapeutic targets. Functional annotation of these genes, through GO and KEGG pathway enrichment analyses, revealed their central roles in immune activation pathways, including “Regulation of T cell activation,” “Leukocyte cell-cell adhesion,” “Allograft rejection,” and “Graft-versus-host disease.” Notably, these pathways are essential mediators of rejection and underpin many of the immune dysregulation processes involved in TCMR. This organ-agnostic approach reinforces the biological relevance of TCMR-Cs and opens the possibility of targeting shared molecular processes across multiple organ transplant types, thereby advancing the development of generalized therapeutic strategies.

Key cytokines within our gene set emerged as significant contributors to TCMR pathogenesis, emphasizing their potential as biomarkers and therapeutic targets. *CXCL13*, for instance, promotes B cell infiltration in kidney transplantation, increasing the severity of rejection, and its elevated levels in serum have been proposed as both a biomarker and therapeutic target for TCMR (30). Similarly, *IFNG* plays a pivotal role in modulating endothelial cell activity and antigen presentation pathways, exacerbating rejection (31). These mechanistic insights into cytokine-mediated immune regulation further validate their potential as molecular targets for therapeutic intervention in TCMR. Other cytokines, such as *CCL3*, *TNFSF13B*, and *CCL18*, are equally important in TCMR. Elevated levels of *CCL3* have been linked to immune cell recruitment and activation in both antibody-mediated rejection (ABMR) and TCMR, indicating its dual relevance (32). Likewise, *TNFSF13B*, which encodes the cytokine BAFF, plays a regulatory role in B-cell activity and rejection-related immune networks (33, 34). Although its direct role in TCMR requires further study, its centrality in autoimmune and rejection pathways highlights its therapeutic potential. Finally, *CCL18* has been implicated in accelerating graft rejection by recruiting alloreactive T cells, as shown in a humanized skin transplant model (35). Taken together, these findings establish a shared cytokine-mediated axis of immune dysregulation that is pivotal to TCMR across multiple organ systems, further validating the importance of our identified gene set as both a biological and therapeutic resource.

Our predictive model, developed using Lasso regression and multivariate logistic regression analysis, demonstrated robust performance, achieving high AUC values in both the training and testing cohorts. These results not only validate the clinical utility of the TCMR-Cs as a robust biomarker but also emphasize the model’s reliability in stratifying TCMR patients based on gene expression profiles. By enabling the early recognition and risk stratification of high-risk patients, this model provides a potential tool for optimizing clinical immunosuppressive regimens, which could ultimately reduce the incidence of acute rejection and improve long-term transplant outcomes. By enabling the early identification of high-risk TCMR patients, the model provides a valuable tool for clinicians to optimize immunosuppressive regimens and potentially reduce the incidence of acute rejection. Compared to previous studies that predominantly focus on single-organ systems (36–38), our multi-organ approach offers a broader perspective by identifying universal biomarkers and therapeutic targets shared across different organ transplant types. This comprehensive strategy underscores the potential for developing organ-agnostic therapeutic interventions.

Further analysis of immune infiltration patterns in TCMR patients revealed significant increases in CD8^+^ T cells, activated natural killer (NK) cells, and M1 macrophages. These immune subsets are key mediators of the effector phase of rejection, contributing to direct cytotoxicity and amplifying inflammatory cascades. The observed enrichment of these immune cell populations aligns with previous studies emphasizing the central role of cellular immunity in TCMR pathogenesis (39–43). These findings provide further evidence that cellular immunity plays a pivotal role in TCMR and highlight the importance of targeting these immune subsets or their upstream pathways to mitigate rejection.

In addition, drug screening through the CMap database identified peroxisome PPARγ agonists as promising candidates for TCMR management. PPARγ agonists are well-known regulators of glucose and lipid metabolism (15), with established clinical applications in treating metabolic disorders such as type 2 diabetes, dyslipidemia, and non-alcoholic fatty liver disease (44). Emerging evidence also suggests that PPARγ agonists exert immunomodulatory effects on T cells by inhibiting the differentiation of Th1, Th2, and Th17 effector T cells, thereby reducing the secretion of associated cytokines such as IFN-γ, IL-4, IL-13, and IL-17A (45, 46). Additionally, PPARγ agonists enhance the generation and functionality of regulatory T cells (Tregs), which play a critical role in suppressing effector T cell activity (46). These mechanisms suggest that PPARγ agonists may have potential as immunomodulatory agents in transplantation medicine. In our study, the PPARγ agonist rosiglitazone demonstrated the ability to suppress T cell activation and IL-2 production *in vitro* and significantly prolonged graft survival *in vivo* when combined with rapamycin. These findings are consistent with the reported anti-inflammatory properties of PPARγ agonists (47–49), supporting their potential as adjunctive therapies in TCMR. However, the mechanisms underlying the synergy between rosiglitazone and rapamycin warrant further investigation.

Despite these promising findings, this study has several limitations. First, the data used to develop the predictive model and identify therapeutic candidates were derived from publicly available databases, which may introduce variability due to differences in data collection and processing methods. Second, the heterogeneity of transplant types and patient populations poses challenges to the generalizability of the model across all clinical scenarios. Future investigations should focus on expanding the sample size and diversity of patient cohorts, particularly by incorporating longitudinal data to assess temporal changes in TCMR-related gene expression and immune infiltration. Finally, while PPARγ agonist rosiglitazone demonstrated immunomodulatory potential in preliminary analyses, its efficacy in clinical settings requires validation through larger *in vivo* and clinical trials. These efforts are essential for optimizing drug dosing strategies, minimizing potential off-target effects, and evaluating combinatorial therapies with existing immunosuppressive regimens.

## 5 Conclusion

In this study, we defined a novel gene set—TCMR-Cs by analyzing gene expression profiles from kidney, heart, and lung transplant biopsies. This gene set formed the basis for a predictive model that demonstrated high diagnostic accuracy across multiple transplant cohorts, validating its potential as a tool for risk stratification and clinical decision-making. Furthermore, using the TCMR-Cs, we identified PPARγ agonists through CMap-based drug repurposing as promising therapeutic candidates for TCMR. Experimental validation showed that PPARγ agonist rosiglitazone effectively suppressed T cell activation *in vitro* and prolonged graft survival *in vivo* when combined with rapamycin. These findings emphasize the importance of TCMR-Cs in understanding cytokine-driven immune dysregulation and highlight the potential of PPARγ agonists as adjunctive therapies in transplantation medicine.

## Data Availability

The original contributions presented in the study are included in the article/**Supplementary Material**. Further inquiries can be directed to the corresponding authors.
