# Supplementary material for: Targeting TCMR-associated cytokine genes for drug screening identifies PPARγ agonists as novel immunomodulatory agents in transplantation
Source: Front Immunol. 2025 Jan 22;16:1539645. doi: 10.3389/fimmu.2025.1539645 (PMC11794815; doi:10.3389/fimmu.2025.1539645)
Supplement: Supplementary file 1 [file DataSheet1.docx]

Supplementary Material

1. **Supplementary Figures**


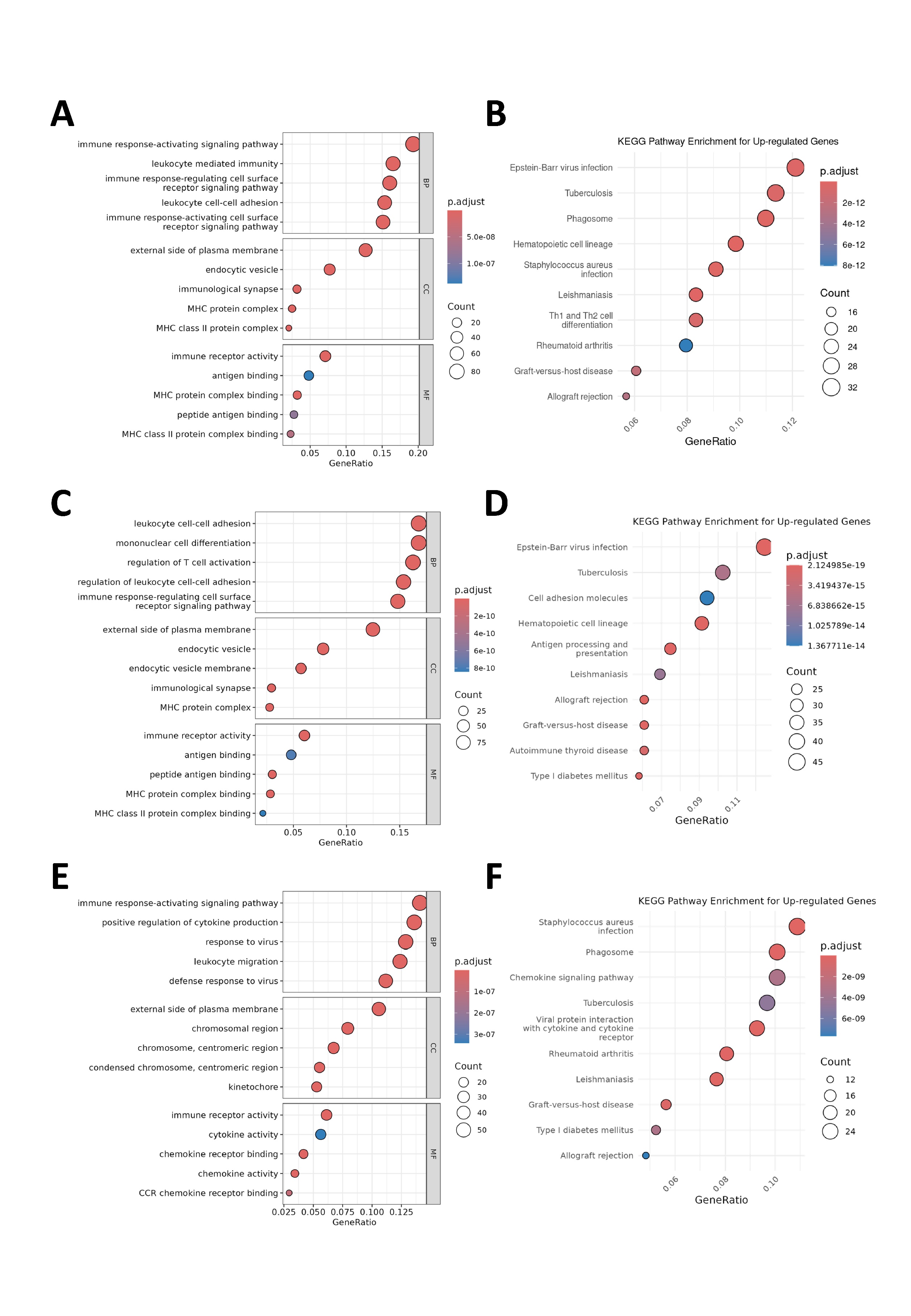


**Supplementary Figure 1.** GO and KEGG functional enrichment analyses of upregulated genes in the TCMR group across different transplant cohorts. Bubble plots display the results of GO functional enrichment analysis (A) and KEGG pathway enrichment analysis (B) for upregulated genes in the TCMR group of the renal transplant cohort (GSE192444). Similarly, GO enrichment (C) and KEGG enrichment (D) results are shown for the heart transplant cohort (GSE150059), and GO enrichment (E) and KEGG enrichment (F) results are presented for the lung transplant cohort (GSE150156).


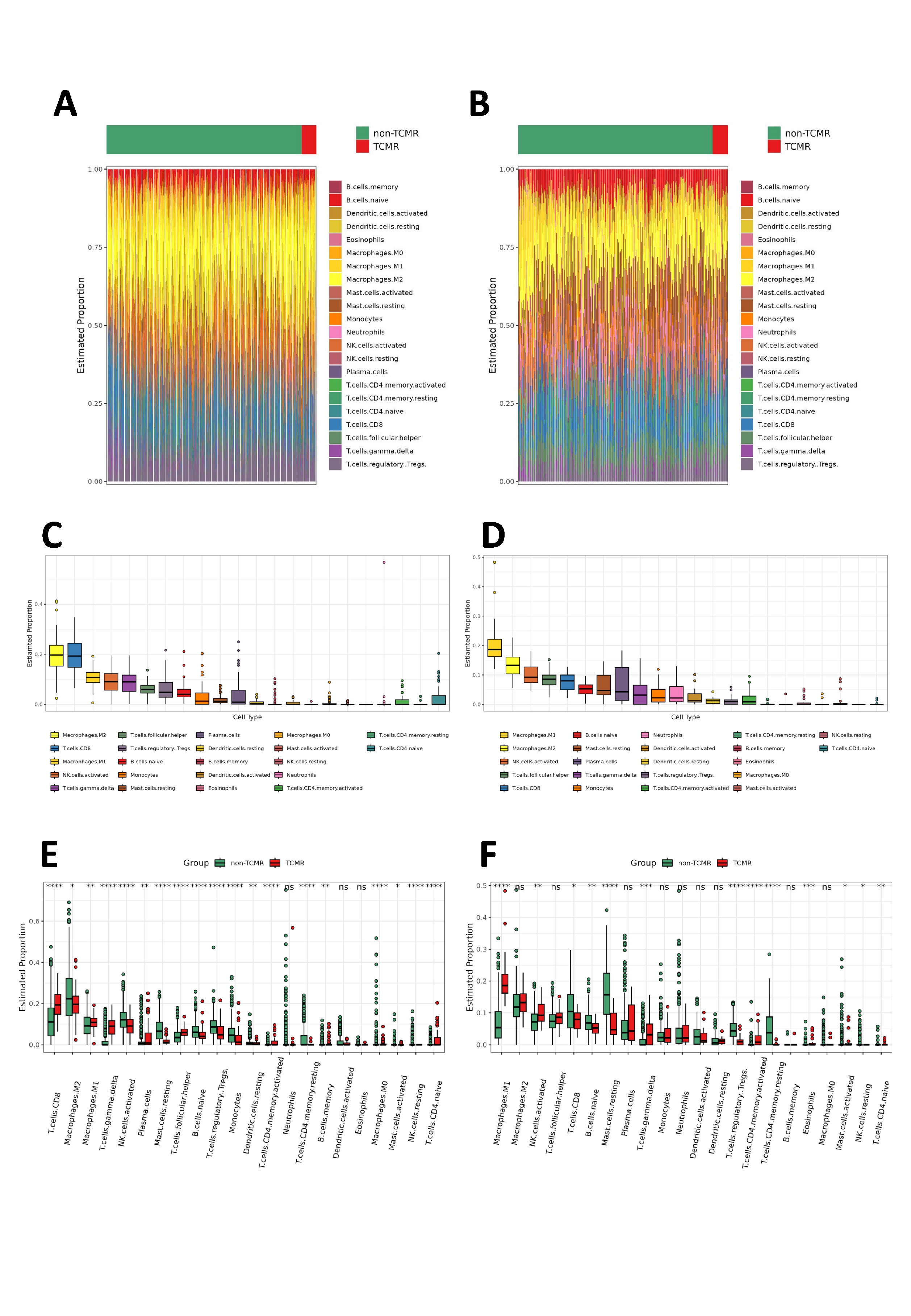


**Supplementary Figure 2.** Immune infiltration characteristics of TCMR patients in heart and lung transplant cohorts. Heatmaps show the infiltration levels of various immune cells in the TCMR and non-TCMR groups within the heart transplant cohort (GSE150059, A) and lung transplant cohort (GSE150156, B). Box plots rank the abundance of immune cell infiltration within the TCMR group in the heart transplant cohort (GSE150059, C) and lung transplant cohort (GSE150156, D). Box plots compare the infiltration levels of immune cells between the TCMR and non-TCMR groups in the heart transplant cohort (GSE150059, E) and lung transplant cohort (GSE150156, F).


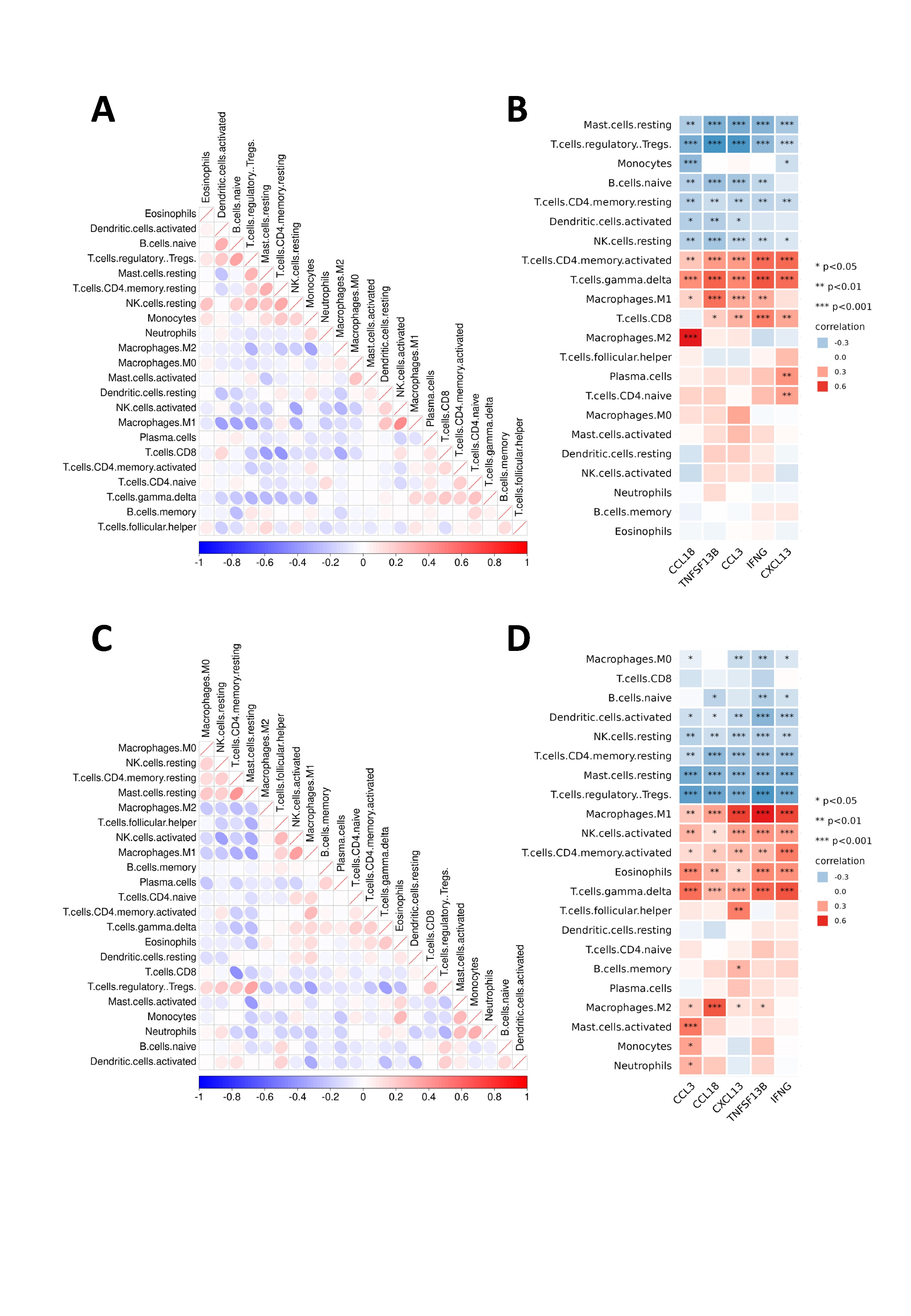


**Supplementary Figure 3.** Correlation analysis of immune infiltration in TCMR patients within heart and lung transplant cohorts. (A, C) Correlation matrices showing relationships among different immune cells in the TCMR group from the heart transplant cohort (GSE150059, A) and lung transplant cohort (GSE150156, C). Blue represents negative correlations, red represents positive correlations, and deeper colors indicate stronger correlation strengths. (B, D) Correlation analysis between TCMR-Hubs and immune cell infiltration levels in the TCMR group from the heart transplant cohort (GSE150059, B) and lung transplant cohort (GSE150156, D). Blue represents negative correlations, red represents positive correlations, and deeper colors indicate stronger correlation strengths. *P < 0.05, **P < 0.01, ***P < 0.001.


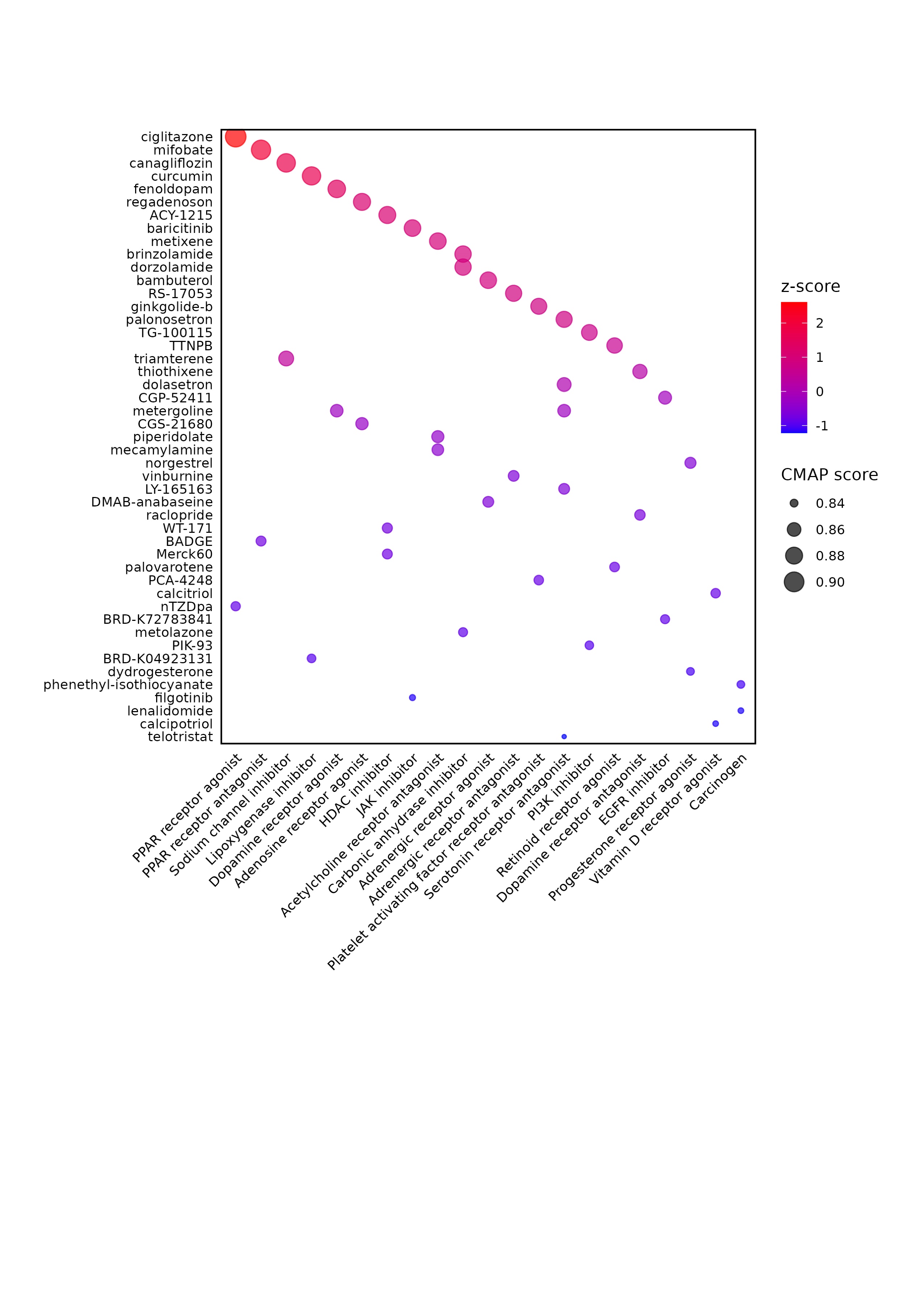


**Supplementary Figure 4.** Bubble plot illustrating potential small-molecule compounds for TCMR treatment, identified from the CMap database. The compounds shown have negative CMap scores, indicating their potential therapeutic effects against TCMR.


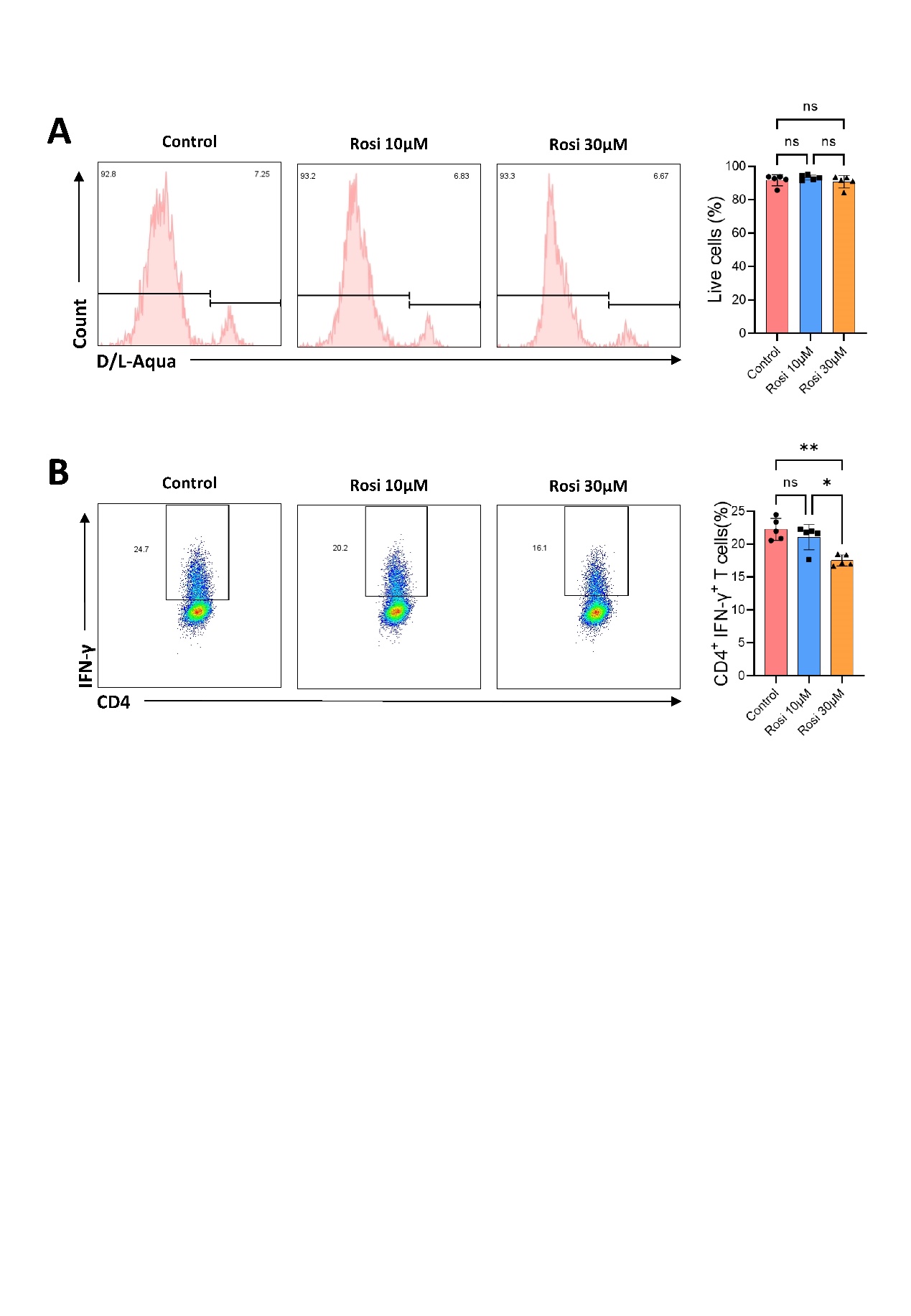


**Supplementary Figure S5.** Effects of rosiglitazone on T cell viability and death, and Th1 differentiation. (A) Cell viability and death of CD4^+^ T cells treated with 10 μM or 30 μM rosiglitazone (Rosi) or an equivalent volume of solvent control for 24 hours. (B) Proportion of Th1 cells (CD4^+^IFN-γ^+^ cells) among CD4^+^ T cells cultured under Th1 differentiation conditions with or without rosiglitazone (Rosi) treatment for 72 hours.
